# Assessing longer-term effectiveness of a combined household-level piped water and sanitation intervention on child diarrhoea, acute respiratory infection, soil-transmitted helminth infection and nutritional status: a matched cohort study in rural Odisha, India

**DOI:** 10.1093/ije/dyz157

**Published:** 2019-07-30

**Authors:** Heather Reese, Parimita Routray, Belen Torondel, Sheela S Sinharoy, Samir Mishra, Matthew C Freeman, Howard H Chang, Thomas Clasen

**Affiliations:** 1 Department of Environmental Health, Rollins School of Public Health, Emory University, Atlanta, GA, USA; 2 Environmental Health Group, London School of Hygiene and Tropical Medicine, London, UK; 3 Kalinga Institute of Industrial Technology, Bhubaneswar, India; 4 Department of Biostatistics and Bioinformatics, Rollins School of Public Health, Emory University, Atlanta, GA, USA

**Keywords:** Sanitation, on-premise piped water, diarrhoea, stunting, soil-transmitted helminth infection

## Abstract

**Background:**

Open defecation is widespread in rural India, and few households have piped water connections. While government and other efforts have increased toilet coverage in India, and evaluations found limited immediate impacts on health, longer-term effects have not been rigorously assessed.

**Methods:**

We conducted a matched cohort study to assess the longer-term effectiveness of a combined household-level piped water and sanitation intervention implemented by Gram Vikas (an Indian NGO) in rural Odisha, India. Forty-five intervention villages were randomly selected from a list of those where implementation was previously completed at least 5 years before, and matched to 45 control villages. We conducted surveys and collected stool samples between June 2015 and October 2016 in households with a child <5 years of age (*n* = 2398). Health surveillance included diarrhoea (primary outcome), acute respiratory infection (ARI), soil-transmitted helminth infection, and anthropometry.

**Results:**

Intervention villages had higher improved toilet coverage (85% vs 18%), and increased toilet use by adults (74% vs 13%) and child faeces disposal (35% vs 6%) compared with control villages. There was no intervention association with diarrhoea [adjusted OR (aOR): 0.94, 95% confidence interval (CI): 0.74–1.20] or ARI. Compared with controls, children in intervention villages had lower helminth infection (aOR: 0.44, 95% CI: 0.18, 1.00) and improved height-for-age z scores (HAZ) (+0.17, 95% CI: 0.03–0.31).

**Conclusions:**

This combined intervention, where household water connections were contingent on community-wide household toilet construction, was associated with improved HAZ, and reduced soil-transmitted helminth (STH) infection, though not reduced diarrhoea or ARI. Further research should explore the mechanism through which these heterogenous effects on health may occur.

**Key Messages**

- An intervention where on-premise piped water coverage was contingent on full community sanitation coverage was associated with improvements in infrastructure coverage and use several years after implementation.
- Although there was no evidence the intervention impacted acute conditions such as diarrhoeal disease or respiratory infection, it was associated with a reduction in soil-transmitted helminth infection and improvements in height-for-age that may require longer-term reductions in faecal exposure.
- The matched-cohort study design and the time lag between intervention implementation and evaluation allowed for assessment of longer-term effects, including time for children to be born into the potentially less contaminated environment and benefit from birth.

## Introduction

Globally, over 2.4 billion people lack access to improved sanitation, and almost one billion people practice open defecation—over half of whom reside in India.^1^ Efforts to address these massive sanitation shortfalls have primarily focused on construction of pour-flush toilets for selected households within communities. The government of India has implemented a succession of large-scale sanitation campaigns across the country.^2^ With a focus on reducing open defecation, however, these efforts emphasized toilet construction at the possible expense of sustained coverage and use.^3^ Health evaluations of these programmes have shown limited impact, possibly due to sub-optimal increases in community-level sanitation coverage and use.^2^^,^^4^^,^^5^

The primary purpose of establishing safe water and improved sanitation is to limit exposure to enteric pathogens associated with a range of poor health outcomes, including diarrhoeal diseases and soil-transmitted helminth (STH) infection.^6–9^ Improved access to water can also increase the quantity available for personal hygiene, which is associated with reduced risk of respiratory infections.^10^^,^^11^ Poor nutritional outcomes are also linked with enteric pathogen exposure, with both underweight and stunting associated with poor household and community-level sanitation.^12–14^ In India, almost half of children <5 years of age are stunted or severely stunted.^15^

Coverage of improved community water sources is relatively high in rural India, but may not be sufficient for flushing or post-defecation cleansing.^1^ While combined water and sanitation interventions have shown limited additive benefits, provision of household piped water, in addition to sanitation, may prove important in increasing use of pour-flush toilets as well as improving water quality for drinking.^16^^,^^17^ However, research on the effects of piped water access on the household premises in combination with sanitation in a rural low-resource context is lacking.

Our objective was to assess the effectiveness of a community-level combined household piped water and sanitation intervention in Odisha, India at least 5 years after intervention completion.

## Methods

### The intervention

The MANTRA program (Movement and Action Network for the Transformation of Rural Areas) was developed by Gram Vikas an Indian non-governmental organization (NGO).^18^ It consists of: (i) a household pour-flush toilet with dual soak-away pits, (ii) an attached bathing room, and (iii) household piped water connections in the toilet, bathing room, and kitchen.^18^ Importantly, for a village to be eligible for participation, every household must commit to constructing their own toilet and bathing room, in addition to other NGO requirements. Gram Vikas assists with the development of a piped water system, which is connected once every household has completed toilet construction. The village is responsible for ongoing costs of operation and maintenance.

### Study design and participants

We used a matched cohort design to assess the longer-term impacts of this previously completed intervention.^19^ We randomly selected 45 villages from a list provided by Gram Vikas of villages with completed interventions in Ganjam and Gajapati districts, Odisha, India, after restriction to those with an intervention start date of 2003–2006. The intervention takes an average of 3 years and the last study village completed implementation in 2010. Forty-five control villages were matched to the 45 intervention villages through a multi-step restriction, matching, and exclusion process to reduce potential bias due to baseline differences.^18^^,^^20^ We used an iterative multivariate matching scheme (R Matching package, version 4.9–2) to match villages on pre-intervention characteristics from the Government of India Census 2001 and Below Poverty Line Survey 2002; balance was achieved on all variables.^18^

Using Monte Carlo simulation to estimate the log odds of child diarrhoeal disease (the primary outcome) we determined a sample size of 45 villages per study arm and 26 children per village, assuming 8.8% diarrhoea prevalence, 0.20 effect size, 80% power, 0.05 significance level and 10% loss to follow-up, as previously reported.^18^

Households with a child <5 years of age at any time during surveillance were eligible for enrollment, and no children aged out of the cohort. In each village, we enrolled up to 40 eligible households, and if more were eligible, we systematically randomly selected 40 across the village. The male and/or female household head provided written informed consent for the household.

### Procedures and outcome measures

Field workers collected data in four rounds approximately every 4 months from June 2015 to October 2016, with household surveys administered to the primary caregiver in the Odia language. For each of the following, each household member reported his own disease status over the previous 7 days, with the caregiver reporting disease for children.^21^ Diarrhoeal disease was defined as at least one occasion of three or more loose stools in the previous 24 h.^19^ Acute respiratory infection (ARI) was defined as cough and/or shortness of breath/difficulty breathing due to chest congestion.^24^ Both diarrhoeal disease and ARI details were collected every study round. Prevalence of bruising or scrapes (combined) was collected in round 3 (February–June 2016) as a negative control to allow qualitative assessment of differential reporting bias for self-reported outcomes.^25^

We used direct observation to assess water, santitation and hygiene (WaSH) infrastructure characteristics. We defined improved sanitation, improved water sources, and presence of a handwashing station (a designated location with water and a cleansing agent present), according to Joint Monitoring Programme standard definitions.^1^ We collected reported interruptions in the preferred drinking water source as: 1) source unavailable for ≥24 h in the previous two weeks, and 2) source unavailable at any time in the previous 24 h. The first measure was collected in all rounds, and the second starting round 2. Interruption in water source was categorized as any interruption, using either measure, across all rounds. Usual defecation location was self-reported for the following categories within each household: elders ≥60 years, men 18–59 years, women 18–59 years, and children 5–17 years. For children <5 years old, the caregiver reported the disposal location for the last defecation event, and improved child faeces disposal was defined as disposal into an improved toilet. We calculated household sanitation use as the proportion of household members each round who reported improved toilet use for defecation (members >5 years old) or for child faeces disposal (members <5 years old), out of the total number of members within each household.

We collected anthropometric measurements for children <5 years old during round 3 (February–June 2016), according to WHO standard methods.^26^^,^^27^ Field workers measured recumbent length for children <2 years old, standing height for children 2–5 years old, and weight for children <5 years old. Height/length were collected in duplicate, and if measurements differed by more than 0.7 cm, a third was collected; the mean of measurements was used to calculate z-scores according to WHO 2006 growth standards (R igrowup macro).^28^ Back-checks on height/length were conducted on a randomly selected 10% of households.

Field workers collected stool samples in round 2 (October 2015–January 2016) from all household members in a randomly selected subset of 500 households to assess the prevalence of common STHs. We used formol ether concentration to quantify worms and ova for hookworms (*Ancylostoma duodenale* and *Necator americanus*), *Ascaris lumbricoides*, *Hymenolepis nana*, and *Tricuris trichura.*^29^^,^^30^ Three slides were examined per sample, with all positives and 10% of negatives examined in duplicate. The mean of measurements was used to estimate eggs per gram of faeces and to quantify worm burden.^29^

### Statistical analysis

We used multilevel logistic regression to estimate intervention association with prevalence of diarrhoeal disease, ARI, bruising/scrapes, and STH infection, and multilevel linear regression to estimate association with height-for-age z score (HAZ), weight-for-age z score (WAZ), and weight-for-height z score (WHZ). Health outcomes measured across all four study rounds, and assessed for all household members during a single round, included random effects for village and household levels to account for repeated measures, and outcomes measured during a single round included a random effect for village level. Profile likelihood confidence intervals (CIs) were estimated to limit potential bias from assumptions of asymptotic normality.

Patterns of missing household-level covariate data were similar across study arms and were handled with multi-level multiple imputation (R pan, version 1.4, and mitml, version 0.3–4, packages).^31^^,^^32^ There was little missing individual-level covariate data; therefore, imputation was restricted to household-level covariates. The imputation model was run for 20 iterations, included all household-level covariates included in regression models, and was adjusted for clustering at the village level. Imputations were used in all subsequent analyses.^33^

We used principal components analysis (R psych package, version 1.6.12) to construct a household wealth index from 15 variables, including household asset ownership, housing characteristics, agricultural land acreage, and below poverty-line status.^34^^,^^35^ We extracted the component that explained the most variability as the wealth index.^36^

Adjusted models were fit with an a priori determined set of covariates to adjust for potential confounding, including the individual’s age and sex, household wealth, religion, caste/tribe status, head of household’s education, primary caregiver’s education, and village access-road quality. Outcomes measured across multiple rounds also included the month of data collection. As sensitivity checks, all regressions were repeated including the village matched pair as a random effect, and all regressions were repeated using the original, unimputed data (see Supplementary Material and Supplementary Table S1 for additional details, available as Supplementary data at *IJE* online). All analyses were completed in R (version 3.3.2).

### Deviations from the study protocol

Outcomes and methods were prespecified, with the following exceptions.^18^ Undernutrition was assessed in children <2 years old in addition to the targeted children <5 years old, to allow comparison with similar studies. Although we intended to assess STH reinfection by collecting a follow-up sample in round 4, this was dropped due to the low stool collection rate in round 2 (75% after two visits) and low STH prevalence.

## Results

### Characteristics of the study population

**Figure 1.**
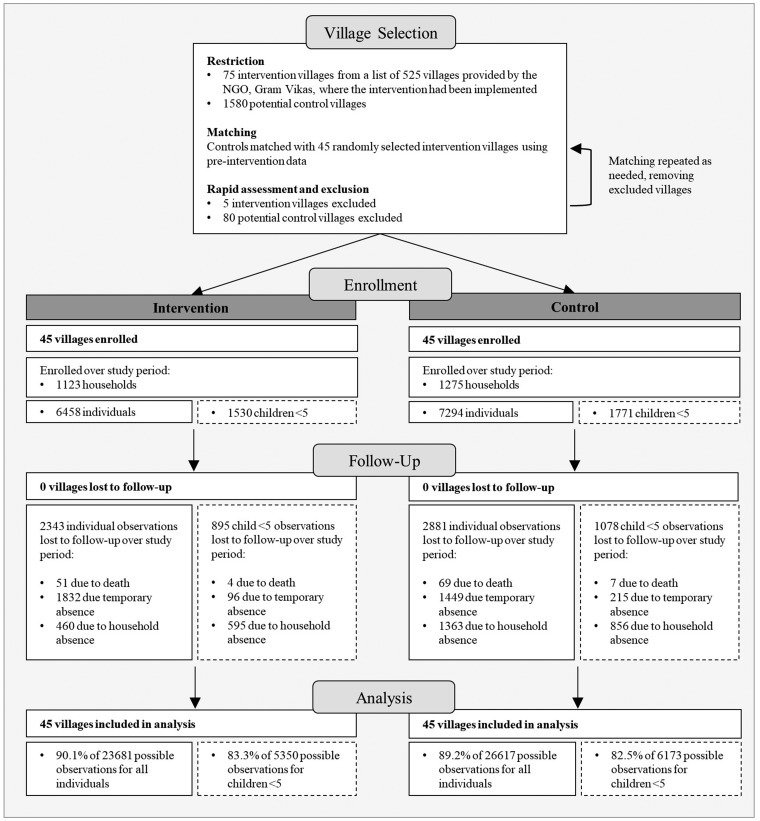
Village selection and profile of the study population across four rounds of data collection. The total number of individuals included at each stage of enrollment, follow-up and analysis is on the left in the intervention and control columns. The subset of the total population that is <5 years old is on the right in dashed boxes.

A total of 1123 households in the intervention villages, and 1275 households in the control villages were enrolled over the four study rounds (Figure 1). An average of 26.5 (range: 2–67) child observations per village per round were available and included in analyses. At follow-up, sociodemographic characteristics were generally similar across study arms, though intervention households were less poor (Table 1).

**Table 1.** Sociodemographic characteristics of the study population, included as covariates in adjusted models

	Control% (*n*)	Intervention% (*n*)	*P*-value
Village characteristics	*n*=45	*n*=45	
Village size (households), $\overset{-}{x}\;$(sd)^b^	157.3 (135.0)	124.0 (92.5)	0.176
Access road paved	91.1% (45)	88.9% (45)	0.726
Household characteristics	*n*=1275	*n*=1123	
Caregiver education ≥5 years	48.0% (612)	57.0% (640)	0.102
Head of household education ≥5 years	38.0% (485)	42.3% (475)	0.203
Caste/tribe			0.147
Scheduled caste	23.7% (255)	13.6% (133)	
Scheduled tribe	15.0% (161)	12.2% (120)	
Other backward caste	39.7% (426)	41.5% (407)	
Other caste	21.6% (232)	32.7% (321)	
Religion			0.632
Hindu	98.8% (1035)	96.7% (902)	
Christian	1.2% (13)	2.8% (26)	
Other	0% (0)	0.5% (5)	
Standardized wealth index, $\overset{-}{x}$ (sd)	0.8 (0.46)	1.0 (0.46)	0.026
Wealth quintile^a^^,b^			0.015
Poorest	25.3% (233)	14.9% (125)	
Poor	20.3% (187)	19.2% (162)	
Middle	20.6% (190)	19.4% (163)	
Rich	18.0% (166)	22.5% (189)	
Richest	15.8% (146)	24.1% (203)	
Individual characteristics	*n*=7395 all ages *n*=1797 children <5 years	*n*=6357 all ages *n*=1502 children <5 years	
Sex, female (all ages)	52.3% (3802)	52.0% (3345)	0.719
Sex, female (children <5 years)	49.0% (860)	49.2% (748)	0.887
Age, years (all ages) $\overset{-}{x}\;$(sd)	24.2 (20.43)	25.0 (20.60)	0.082
Age, months (children <5) $\overset{-}{x}$ (sd)	28.5 (17.72)	29.4 (17.68)	0.218

Wald *P*-values are adjusted for clustering at village level for household characteristics, and at village and household levels for individual characteristics. sd = standard deviation.

a Wealth quintile captures the proportion of households in each quintile of the standardized wealth index.

b Not included as a covariate in adjusted models. Provided here for descriptive purposes.

### Coverage, access and use of water, hygiene and sanitation facilities

Access to a household improved toilet was almost five times higher in intervention than control villages (85.0% vs 17.7%; Table 2). Coverage of household piped water for both drinking and other purposes, including cooking, hygiene, and toilet flushing, as well as presence of a functional hand-washing station was substantially higher in the intervention than control arm. The intervention was positively associated with minor improvements in round-trip time to water source, though with higher prevalence of water intermittency, likely due to greater reliance on the piped system in the intervention arm. The proportion of household members using improved sanitation for defecation was also substantially higher in intervention than control villages (59.3% vs 12.9% of members), with almost all remaining members reporting open defecation (Table 2).

**Table 2.** Household water, sanitation and hygiene coverage, access and use characteristics across all study rounds, unless otherwise noted

	*n*	Control% (*n*)	Intervention% (*n*)	*P*-value
Water, sanitation and hygiene coverage				
Improved toilet^a^	2105	17.7% (198)	85.0% (837)	<0.001
Toilet with soak-away/septic tank	2105	17.3% (194)	78.4% (772)	<0.001
Improved drinking water source^a^	2388	72.0% (913)	92.1% (1031)	<0.001
Household piped water^a^	2388	8.0% (102)	72.7% (813)	<0.001
Improved water source for other purposes^a^	2110	62.9% (707)	90.1% (888)	<0.001
Household piped water^a^	2110	8.3% (93)	73.3% (723)	<0.001
Hand-washing station	6048	61.7% (1934)	85.3% (2487)	<0.001
Water available	7529	61.5% (2409)	83.1% (2998)	<0.001
Soap/detergent available	7528	25.1% (982)	48.9% (1764)	<0.001
Ash/sand available	7528	37.3% (1463)	27.2% (981)	<0.001
Bathing room	1902	12.1% (121)	82.1% (739)	<0.001
Water access				
Interruption in water availability, any	7807	7.1% (291)	16.5% (609)	<0.001
Anytime in previous 24 h^b^	7806	4.3% (177)	9.5% (353)	<0.001
≥24 h in previous two weeks	3888	6.4% (198)	15.2% (421)	<0.001
Time to water source (min), $\overset{-}{x}$ (sd)	5766	10.2 (11.5)	3.5 (6.7)	<0.001
Water storage, any	7805	99.5% (4099)	97.7% (3601)	<0.001
Water storage, safe	7786	20.6% (849)	22.6% (831)	<0.001
Narrow-mouthed container (<6 cm)	7681	24.7% (1009)	26.0% (913)	<0.001
Covered container	7682	83.0% (3398)	86.2% (3094)	<0.001
Improved sanitation use				
Proportion of household using, all ages, $\overset{-}{x}$ (sd)	5890	12.9% (28.8%)	59.3% (36.0%)	<0.001
Toilet use, ≥60 years	3023	17.8% (279)	76.2% (1107)	<0.001
Toilet use, men 18–59 years	5395	15.0% (428)	74.5% (1900)	<0.001
Toilet use, women 18–59 years	5833	18.2% (561)	79.5% (2182)	<0.001
Toilet use, 5–17 years	3904	16.8% (351)	76.4% (1387)	<0.001
Child faeces disposal, <5 years	5367	8.8% (250)	39.2% (989)	<0.001

*P*-values are adjusted for clustering at village level. sd = standard deviation.

a Reported once for each household.

b Data available rounds 2–4.

### Health outcomes

Prevalence of 7-day diarrhoea in children <5 years old, the primary study outcome, and 7-day prevalence of ARI were similar across intervention and control villages (5.3 vs 4.9%, and 9.3 vs 10.3%; Table 3). Prevalence of any STH infection among children was almost twice as high in control villages as in intervention (6.8 vs 3.9%; Table 3). No *A. lumbricoides* and few *T. trichiura* infections were found in either study arm; the helminth burden was primarily due to infection with hookworms or *H. nana*. A smaller proportion of children <5 years old were stunted (33.3 vs 40.4%), wasted (10.3 vs 12.3%) or underweight (26.5 vs 34.8%) in intervention villages compared with control (Table 3).

**Table 3.** Prevalence of health outcomes in children <2 years old, children <5 years old, and all household members. Prevalence across all study rounds is shown for self-reported health, prevalence at round 2 (Oct 2015–Jan 2016) is shown for STH infection, and prevalence at round 3 (Feb–June 2016) is shown for nutrition and control outcomes

	*n*	Control % (*n*)	Intervention% (*n*)	*P*-value
Children <5 years old				
Self-reported health				
Diarrhoea	8875	5.3% (251)	4.9% (199)	0.557
Acute respiratory infection	8964	9.3% (127)	10.3% (122)	0.959
Soil-transmitted helminth infection				
Any STH prevalence	775	6.8% (28)	3.9% (14)	0.044
*Ascaris lumbricoides* prevalence	775	0.0% (0)	0.0% (0)	1.000
*Trichuris trichiura* prevalence	775	0.0% (0)	0.0% (0)	1.000
*Hymenolepis nana* prevalence	775	1.5% (6)	1.1% (4)	0.659
*Hymenolepis nana* intensity (epg), $\overset{-}{x}$ (sd)	775	2.4 (13.72)	1.1 (9.32)	0.270
Hookworm prevalence	775	5.3% (22)	2.8% (10)	0.095
Hookworm intensity (epg), $\overset{-}{x}$ (sd)	775	1.8 (24.04)	0.4 (3.62)	0.115
Nutrition outcomes				
HAZ, $\overset{-}{x}$ (sd)	1826	−1.77 (1.12)	−1.48 (1.17)	<0.001
Stunted (HAZ<-2)	1826	40.4% (402)	33.3% (277)	0.063
Severely stunted (HAZ<-3)	1826	14.0% (139)	7.9% (66)	0.356
WAZ, $\overset{-}{x}$ (sd)	1893	−1.61(1.08)	−1.36 (1.11)	0.019
Underweight (WAZ<-2)	1893	34.8% (362)	26.5% (226)	0.030
Severely underweight (WAZ<-3)	1893	9.8% (102)	6.2% (53)	0.602
WHZ, $\overset{-}{x}$ (sd)	1829	−0.85 (1.03)	−0.75 (1.06)	0.146
Wasted (WHZ<-2)	1829	12.3% (123)	10.3% (86)	0.808
Severely wasted (WHZ<-3)	1829	1.5% (15)	1.0% (8)	0.303
Control				
Bruising/scrapes	2172	3.8% (45)	3.5% (35)	0.738
Children <2 years old				
Nutrition outcomes				
HAZ, $\overset{-}{x}$ (sd)	655	−1.67 (1.20)	−1.35 (1.33)	0.013
Stunted (HAZ<-2)	655	38.0% (136)	30.0% (89)	0.070
Severely stunted (HAZ<-3)	655	15.1% (54)	9.1% (27)	0.311
WAZ, $\overset{-}{x}$ (sd)	685	−1.49 (1.11)	−1.21(1.22)	0.038
Underweight (WAZ<-2)	685	30.3% (115)	21.6% (66)	0.054
Severely underweight (WAZ<-3)	685	10.3% (39)	5.9% (18)	0.384
WHZ, $\overset{-}{x}$ (sd)	659	−0.76 (1.09)	−0.67 (1.05)	0.244
Wasted (WHZ<-2)	659	12.2% (44)	8.4% (25)	0.413
Severely wasted (WHZ<-3)	659	1.7% (6)	0.7% (2)	0.130
All household members				
Self-reported health				
Diarrhoea	40436	2.8% (593)	2.4% (485)	0.092
Acute respiratory infection	40999	4.3% (254)	6.6% (241)	0.678
Soil-transmitted helminth infection				
Any STH prevalence	1452	11.5% (86)	8.6% (61)	0.273
*Ascaris lumbricoides* prevalence	1452	0.0% (0)	0.0% (0)	1.000
*Trichuris trichiura* prevalence	1452	0.0% (0)	0.0% (1)	0.997
*Trichuris trichiura* intensity (epg), $\overset{-}{x}$ (sd)	1452	0.0 (0)	0.0 (0.1)	0.318
*Hymenolepis nana* prevalence	1452	1.9% (14)	1.6% (11)	0.714
*Hymenolepis nana* intensity (epg), $\overset{-}{x}$ (sd)	1452	3.8 (66.2)	0.78 (9.7)	0.238
Hookworm prevalence	1452	9.7% (72)	7.2% (51)	0.366
Hookworm intensity (epg), $\overset{-}{x}$ (sd)	1452	5.8 (24.2)	3.7 (18.4)	0.333
Control				
Bruising/scrapes	10091	1.7% (93)	1.5% (70)	0.276

*P*-values adjusted for clustering at village and household levels. sd = standard deviation.

There was no intervention association with 7-day diarrhoea prevalence for children <5 years old (adjusted OR (aOR): 0.98, 95% CI: 0.77, 1.25) or with 7-day ARI prevalence (aOR: 1.03, 95% CI: 0.84–1.25; Table 4). There was also no intervention effect on prevalence of bruising/scrapes, collected as a negative control for self-reported health outcomes. However, there was evidence that the intervention had a protective effect on infection with any STH in children (aOR: 0.44, 95% CI: 0.18, 1.00); though not in all household members (Table 4). The intervention was positively associated with increased HAZ in children <5 years old (+0.17 HAZ, 95% CI: 0.03, 0.31) (Table 4). The association between the intervention and HAZ in children <2 years old was similar in magnitude to that in children <5 years old, but was not as strong. This may be due to not being sufficiently powered to detect an effect in the child <2 years age group. There was no intervention association with either WAZ or WHZ (Table 4).

**Table 4.** Effect of the intervention on health in children <2 years old, children <5 years old, and all household members

		Unadjusted		Adjusted	
	*n*	OR (95% CI)	*P*-value	OR (95% CI)	*P*-value
Children under 5 years					
Self-reported health					
Diarrhoea	8875	0.93 (0.73, 1.18)	0.557	0.98 (0.77, 1.25)	0.855
Acute respiratory infection	8964	1.00 (0.84, 1.18)	0.959	1.03 (0.84, 1.25)	0.363
Soil-transmitted helminth infection					
STH infection, any^b^	777	0.49 (0.20, 1.08)	0.077	0.44 (0.18, 1.00)	0.049
Nutrition outcomes					
Height-for-age z score^a^	1826	0.26 (0.06, 0.46)	0.011	0.17 (0.03, 0.31)	0.015
Weight-for-age z score^a^	1893	0.22 (0.01, 0.42)	0.038	0.13 (-0.01, 0.27)	0.068
Weight-for-height z score^a^	1829	0.08 (-0.07, 0.24)	0.288	0.04 (-0.09, 0.16)	0.587
Control					
Bruising/scrapes	2172	0.93 (0.59, 1.45)	0.737	0.88 (0.55, 1.41)	0.601
Children <2 years					
Nutrition outcomes					
Height-for-age z score^a^	655	0.31 (0.04, 0.57)	0.026	0.17 (-0.04, 0.38)	0.110
Weight-for-age z score^a^	685	0.23 (-0.03, 0.49)	0.077	0.08 (-0.11, 0.28)	0.390
Weight-for-height z score^a^	659	0.07 (-0.13, 0.27)	0.481	0.00 (-0.17, 0.18)	0.958
All household members					
Self-reported health					
Diarrhoea	40409	0.85 (0.72, 1.01)	0.063	0.86 (0.74, 1.03)	0.122
Acute respiratory infection	40999	1.03 (0.90, 1.18)	0.688	1.08 (0.94, 1.24)	0.288
Soil-transmitted helminth infection					
STH infection	1452	0.69 (0.40, 1.16)	0.161	0.72 (0.42, 1.19)	0.192
Control					
Bruising/scrapes	10091	0.89 (0.42, 1.88)	0.764	0.86 (0.41, 1.39)	0.660

a Marginal effect, not odds ratio.

b Household religion excluded from adjusted model due to lack of variability.

## Discussion

To our knowledge, this study is the first evaluating the effectiveness of a combined on-premise water and sanitation intervention in rural India, and the first to assess the longer-term impacts of such an intervention. In contrast to interventions that involve only community water supplies and/or partial community sanitation coverage, the Gram Vikas MANTRA intervention was designed to provide piped water at each home and ensure every household had an improved toilet and bathing room. However, there was no evidence the intervention was protective against diarrhoeal disease, the primary study outcome, or ARI, despite increases in water and hand-washing station coverage. In contrast, our findings suggest the intervention was protective against child STH infection as well as effective in improving HAZ in children <5 years old.

The lack of a protective effect on diarrhoea is consistent with previous evaluations of sanitation interventions in India.^4^^,^^5^^,^^37^ Despite sanitation and hygiene deficiencies, diarrhoea prevalence is comparatively low, providing limited opportunity for improvements. The lack of an association with ARI may be due to continued insults from indoor and ambient air pollution not impacted by this intervention. The protective effect on STH infection is in contrast with previous studies in India, where community sanitation coverage and use was not as high, but consistent with overall evidence on sanitation impacts.^38^^,^^39^

The protective effect of the intervention on HAZ is noteworthy given the high levels of stunting in India and the hypothesis that this may be attributable to environmental enteric dysfunction.^40^ The observed effect was similar in magnitude to that in a previous study with similarly large reductions in reported open defecation within a community-level approach.^13^ However, unlike in this previous study, there was a similar magnitude effect in both children <2 years old and all children <5 years old.^13^ Since our study began years after intervention completion, there was the opportunity for children to be born into potentially less fecally contaminated environments, benefit from the intervention from birth, and thus have sustained nutritional benefits past the key developmental window of 6–24 months. Other recent trials have reported no effect on linear growth from combined WaSH interventions, though these were not community-wide interventions and were implemented in settings where open defecation was uncommon.^41^^,^^42^

Notwithstanding these heterogenous effects on health, there were substantial gains in WaSH coverage, access, and use. In these respects, the intervention was effective in accomplishing the target outputs of many WaSH initiatives. However, intermittent availability of preferred water sources and subsequent high levels of drinking water storage provided a possible source of continued exposure to enteric pathogens. The increase in household piped water coverage may have indirectly impacted child health through increasing toilet use, instead of expected direct impacts if the piped system provided microbiologically high-quality water.

Our study design and methods presented certain limitations. First, as we were interested in assessing longer-term effects, we employed a study design in which the intervention status was not randomly assigned. Although study arms were well balanced at the village-level after matching on available pre-intervention characteristics, we cannot rule out imbalance on unobserved variables and the potential for residual confounding. The intervention involves the commitment and active participation of the entire village, attributes that are difficult to measure, especially retrospectively, and thus balance. In addition, pre-intervention disease prevalence was not available for matching. To limit bias, a set of a priori determined potential confounders were included in all models. The time lapse between intervention completion and study initiation necessitated matching villages on pre-intervention characteristics measured several years prior to the evaluation process, and prevented assessment of immediate impacts. On the other hand, the retrospective design allowed us to assess longer-term impacts, a challenge for experimental designs with limited funding and follow-up. In addition to its policy relevance, this longer-term assessment also provided a biologically plausible length of time for die-off of even the most persistent pathogens in the environment, and time for the target population, children <5 years old, to be born into this environment. Another limitation is that diarrhoeal disease and ARI were collected using self- and caregiver reports—a method that may be subject to measurement bias.^43^^,^^44^ However, we found no effect on our negative control outcome, indicating any potential measurement bias for self-reported health was not differential by study arm. Moreover, we found no protective intervention effects on these reported outcomes, diarrheal disease and ARI. In contrast, we found protective effects on STH infection and anthropometrics, outcomes that were objectively assessed and therefore not susceptible to reporting bias.

Finally, there are limitations to generalizability. Although intervention study villages were randomly selected from those where the implementation was complete, and so results should be representative of those on the list, we understand from Gram Vikas that there are villages that received a motivation visit but declined participation. While we excluded these from the list of potential controls, non-participating villages may be different from participating villages in their awareness of health risks, collective efficacy, or other characteristics. Thus, it should not be assumed that the MANTRA intervention can be successfully implemented across all villages in this setting or elsewhere. Future planned analysis of collective efficacy may shed light on its contribution to programme implementation and effectiveness.

In conclusion, this study provides evidence that a combined intervention, where provision of household piped water connections is contingent on community sanitation coverage, can substantially decrease open defecation. Although we found no evidence these reduced child diarrhoea or ARI, our results suggest a protective effect against STH infection and HAZ. Future planned analyses, including assessment of fecal environmental contamination, environmental enteric dysfunction, and collective efficacy, may provide a fuller understanding of both the biological and behavioural mechanisms for these heterogeneous effects on child health. Given previous evidence that increasing sanitation use, even with high coverage, is especially difficult in rural India, this study provides evidence to support a combined community-level implementation of household piped water and sanitation.^4^^,^^5^^,^^45^

## Funding

This work was supported by Bill & Melinda Gates Foundation [grant numbers OPP1008048 and OOP1125067].

## Supplementary Material

dyz157_Supplementary_Materials
