# Supplementary material for: Assessing longer-term effectiveness of a combined household-level piped water and sanitation intervention on child diarrhoea, acute respiratory infection, soil-transmitted helminth infection and nutritional status: a matched cohort study in rural Odisha, India
Source: Int J Epidemiol. 2019 Jul 30;48(6):1757–67. doi: 10.1093/ije/dyz157 (PMC6929523; doi:10.1093/ije/dyz157)
Supplement: dyz157_Supplementary_Materials [file dyz157_supplementary_materials.docx]

**Supplementary Material**

**Sensitivity analyses**

A sensitivity analysis was conducted to compare the model framework to one which included a random effect for each matched village pair. The results of the matched pair analyses were qualitatively similar, however they appeared to be less conservative, with consistently narrower confidence intervals. Due to this, the intervention was positively associated with weight-for-age z score; however, there were no other meaningful differences. In addition, a sensitivity analysis was conducted comparing use of the multi-level imputed data with the unimputated data containing missing covariate values (Table S1). We found no meaningful differences in adjusted health estimates, with the exception of intervention association with soil-transmitted helminth infection for children under five. Although models for soil-transmitted helminth infection for both children and all household members are not directly comparable due to the necessary exclusion of three covariates from the unimputed models, this difference means these results should be interpreted cautiously.

**Table S1.** Effect of the intervention on health in children under two years, children under five years, and all household members, adjusted using multilevel imputed covariate data (left) and unimputed covariate data (right).

|  |  | **Adjusted Imputed** |  |  | **Adjusted Unimputed** |  |
| --- | --- | --- | --- | --- | --- | --- |
|  | n | **OR (95% CI)** | **p-value** | n | **OR (95% CI)** | **p-value** |
| **Children under 2 years** |  |  |  |  |  |  |
| Nutrition outcomes |  |  |  |  |  |  |
| Height-for-age z score^†^ | 655 | 0.17 (-0.04, 0.38) | 0.110 | 532 | 0.15 (-0.07, 0.37) | 0.205 |
| Weight-for-age z score^†^ | 685 | 0.08 (-0.11, 0.28) | 0.390 | 559 | 0.06 (-0.13, 0.26) | 0.538 |
| Weight-for-height z score^†^ | 659 | 0.00 (-0.17, 0.18) | 0.958 | 536 | -0.01 (-0.18, 0.18) | 0.944 |
| **Children under 5 years** |  |  |  |  |  |  |
| Self-reported health |  |  |  |  |  |  |
| Diarrhea | 8875 | 0.98 (0.77, 1.25) | 0.855 | 6887 | 1.05 (0.80, 1.37) | 0.740 |
| Acute respiratory infection | 8964 | 1.03 (0.84, 1.25) | 0.363 | 6955 | 1.02 (0.84, 1.25) | 0.802 |
| Soil-transmitted helminth infection |  |  |  |  |  |  |
| STH infection, any | 777 | 0.44 (0.18, 1.00)^‡^ | 0.049 | 596 | 0.64 (0.23, 1.65)^§^ | 0.353 |
| Nutrition outcomes |  |  |  |  |  |  |
| Height-for-age z score^†^ | 1826 | 0.17 (0.03, 0.31) | 0.015 | 1551 | 0.14 (0.01, 0.28) | 0.046 |
| Weight-for-age z score^†^ | 1893 | 0.13 (-0.01, 0.27) | 0.068 | 1612 | 0.09 (-0.04, 0.23) | 0.193 |
| Weight-for-height z score^†^ | 1829 | 0.04 (-0.09, 0.16) | 0.587 | 1554 | 0.01 (-0.12, 0.15) | 0.834 |
| Control |  |  |  |  |  |  |
| Bruising/scrapes | 2172 | 0.88 (0.55, 1.41) | 0.601 | 1837 | 1.00 (0.61, 1.64) | 0.988 |
| **All household members** |  |  |  |  |  |  |
| Self-reported health |  |  |  |  |  |  |
| Diarrhea | 40409 | 0.86 (0.74, 1.03) | 0.122 | 32479 | 0.93 (0.77, 1.12) | 0.436 |
| Acute respiratory infection | 40999 | 1.08 (0.94, 1.24) | 0.288 | 32866 | 1.03 (0.91, 1.23) | 0.480 |
| Soil-transmitted helminth infection |  |  |  |  |  |  |
| STH infection | 1452 | 0.72 (0.42, 1.19) | 0.192 | 1195 | 0.74 (0.42, 1.31)^§^ | 0.301 |
| Control |  |  |  |  |  |  |
| Bruising/scrapes | 10091 | 0.86 (0.41, 1.39) | 0.660 | 8872 | 0.99 (0.43, 2.27) | 0.977 |

† Marginal effect, not odds ratio

‡ Household religion excluded from adjusted model due to lack of variability

§ Household religion, caregiver education, and head of household education excluded from adjusted model due to lack of variability or collinearity
